# The Impact of Late Adolescents' Social Network Strength on Mental Health

**DOI:** 10.1002/brb3.70910

**Published:** 2025-09-27

**Authors:** Jin Seok Oh, Jong Gab Ho, Jin‐Hyuck Park

**Affiliations:** ^1^ Soonchunhyang Exceptional Children Institute Soonchunhyang University Asan South Korea; ^2^ Department of Occupational Therapy, College of Medical Science Soonchunhyang University Asan South Korea

**Keywords:** late adolescents, mental health, social network analysis, social network strength

## Abstract

**Purpose:**

This study examined how social network strength (i.e., the intensity and frequency of interactions within one's personal network, assessed using centrality measures such as degree, closeness, betweenness, and eigenvector centrality) affects mental health.

**Methods:**

The study was conducted with 108 first‐year university students. Data were collected using a structured questionnaire, and NetMiner 4.0 was employed to analyze degree centrality, closeness centrality, betweenness centrality, and eigenvector centrality as indicators of social network centrality. Additionally, Quadratic Assignment Procedure (QAP) correlation analysis and QAP regression analysis were conducted to assess the relationship between social network strength and mental health.

**Results:**

The study found that stronger social networks were linked to better mental health in late adolescence. Specifically, higher social network centrality was associated with lower levels of hopelessness and depression. QAP correlation analysis revealed that hopelessness had a significant negative correlation with in‐closeness centrality (*which reflects how easily one can be reached by others within the network*) (*r* = −0.252, *p* < 0.05), while depression was negatively correlated with in‐degree centrality (*the number of direct incoming connections from peers*) (*r* = −0.233, *p* < 0.05), in‐closeness centrality (*r* = −0.256, *p* < 0.05), and eigenvector centrality (*a measure of how well a person is connected to popular or influential members of the network*) (*r* = −0.291, *p* < 0.01). QAP regression analysis further confirmed that weaker social ties, indicated by higher out‐closeness centrality (*how easily one can reach others, especially those at a distance*), were associated with higher depressive symptoms (adj. *R* ^2^ = 0.143, *F* = 2.423, *p* < 0.05). These results suggest that building stronger and more integrated social networks may help reduce psychological distress and support mental well‐being in late adolescence.

**Conclusion:**

Stronger social networks in late adolescence are associated with better mental health, highlighting the importance of social connections. While correlational findings suggest general links between centrality and emotional well‐being, the regression results underscore specific predictors with practical implications. In‐degree, in‐closeness, and eigenvector centrality were associated with reduced depressive symptoms, indicating that being well‐integrated within a social network may offer protective benefits. In contrast, high out‐closeness centrality predicted increased depression, suggesting that the effort required to maintain outward‐reaching connections may impose psychological burdens. These findings suggest that targeted interventions should focus on strengthening inward social integration while addressing the stress related to maintaining extensive outward connections, providing direction for more effective youth mental health strategies.

## Graphical abstract

This study of 108 first‐year university students shows that stronger in‐degree and in‐closeness centrality reduce hopelessness and depression, while higher out‐closeness centrality increases depression.

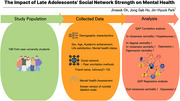

## 1 Introduction

Late adolescence is generally recognized as a transitional stage from adolescence to adulthood, typically encompassing individuals aged 19–24 (Kim and Jeong 2015). During this period, individuals explore and make decisions regarding personal values, career paths, and interpersonal relationships, all of which contribute to the formation of self‐identity (Marcia 1966). Additionally, late adolescence is marked by the development of autonomy, characterized by increased independent decision‐making and responsibility, alongside social role transitions, such as moving from education to employment and experiencing changes in family dynamics (Arnett 2000).

From a cross‐cultural perspective, however, the chronological boundaries of late adolescence differ across countries. The World Health Organization (WHO) defines late adolescence as occurring between ages 19 and 24, emphasizing this period as critical for preparing for adult roles and establishing lifelong health habits (Palenzuela‐Luis et al. 2022). In Belgium, late adolescence is often considered to span ages 15–19 (Van Droogenbroeck et al. 2018), reflecting early psychosocial transitions. In the United States, it typically ranges from 17 to 22 years (Laursen et al. 2017), while in Finland, adolescents aged 16–18 are classified within this phase (Salmela‐Aro et al. 2017). Similarly, Japan often identifies 15‐ to 18‐year‐olds as late adolescents (Yamasaki et al. 2016), particularly within the high school system. These differences highlight how cultural, educational, and institutional contexts shape the operational definition of late adolescence, suggesting the need to interpret this developmental phase within a culturally sensitive framework.

As a result, individuals in this stage often experience significant psychological changes, including heightened concerns about their future and personal identity (Erikson 1968). Although they are expected to assume adult roles and responsibilities, many have yet to achieve full independence, facing challenges related to social expectations, education, employment, and financial stability, which may lead to uncertainty and stress (Arnett 2000). In addition to these psychological and social transformations, mental health issues become increasingly prominent during late adolescence. Research has linked this stage to higher rates of suicidal ideation and mental health disorders.

In South Korea, the suicide rate among late adolescents has shown a continuous increase, reaching 19.6 per 100,000 people in 2023, up from 18.1 in 2022 and 14.5 in 2013 (Statistics Korea [KOSTAT] 2023). To contextualize these figures globally, Japan reported a significantly higher suicide rate for late adolescents, with 354 per 100,000 in 2022 and 351 in 2023 (Statista 2025a). In New Zealand, the rates were 16.9 in 2018, 23.14 in 2019, and 18.69 in 2020 (Statista 2025b). Finland also showed an upward trend, from 24 in 2022 to 45 per 100,000 in 2023 (Finnish Institute for Health and Welfare 2025). Meanwhile, Australia reported 128 per 100,000 in 2022 and 143 in 2023 (Australian Institute of Health and Welfare 2025). These cross‐national comparisons highlight that while South Korea's rate is moderate relative to some countries, it still reflects a concerning upward trend that warrants urgent public health attention.

Globally, suicide rates among individuals in their 20s have also been rising, drawing attention as a critical public health issue. The WHO compiles and publishes annual suicide data by country and age group. According to the 2019 WHO Suicide Worldwide report, 703,000 people die by suicide annually, highlighting suicide as a significant cause of death and an urgent issue requiring global attention (WHO 2024). Given this growing concern, mental health management and suicide prevention strategies for late adolescents are essential. A comprehensive and systematic approach to suicide prevention is crucial in mitigating recurrent suicide attempts and reducing the widespread impact of suicide‐related deaths (WHO 2024).

Since interventions are less effective after a suicide attempt, prevention is the most critical strategy. It is essential to identify at‐risk late adolescents early and develop effective intervention measures (Park 2016). To enhance suicide prevention efforts, society must first recognize the severity of the issue and systematically analyze factors influencing suicidal ideation among late adolescents (Wu and Sheng 2019). Social isolation resulting from various social barriers can significantly impact mental health, increasing the risk of suicidal behavior (Calati et al. 2019). Among university students, these barriers may include academic overload, competitive peer environments, faculty relationship stress, and limited opportunities for social interaction, especially in programs with heavy coursework or relocation away from prior support networks. Moreover, the pressure to present idealized lives on social media can contribute to feelings of inadequacy and disconnectedness (Huang et al. 2023; Jo and Baek 2023; Ray et al. 2019).

These studies have contributed to our understanding of the association between social factors and suicidal ideation. However, the psychosocial mechanisms through which the structure of social networks impacts mental health remain an underexplored domain. Social networks function not merely as structural configurations of interpersonal ties but as conduits for emotional support, recognition, and access to information resources known to buffer psychological distress (Pachucki et al. 2015). For instance, individuals who occupy central positions within social networks are more likely to receive social support and feedback from peers, which may mitigate feelings of isolation and hopelessness (Bringmann et al. 2019).

Furthermore, peer relationships play a pivotal role in identity formation during adolescence (Clarke 2009). Social interactions within networks foster role exploration, self‐esteem development, and a sense of belonging, which are recognized protective factors against depression and suicidal ideation (Jetten et al. 2012). Accordingly, network centrality may serve not only as a structural index but also as a proxy indicator for access to psychosocial resources, offering a meaningful lens through which to evaluate an individual's integration within their social ecology and its implications for mental health.

Previous studies on social networks and suicide have empirically examined the impact of social relationships on suicidal thoughts and behaviors. Some studies have analyzed family effects as both risk and protective factors for suicide (Lee et al. 2010), while others have investigated the moderating role of social networks in the relationship between elder abuse, depression, and suicide risk (Sim and Hong 2019). Additionally, research using social integration surveys has considered how social capital influences suicidal ideation, accounting for differences by age and gender (Chung 2021). Another study explored the sequential mediation effects of future outlook and social isolation on the relationship between relative deprivation and suicidal ideation in young adults (Lee et al. 2022).

However, many existing studies rely on secondary big data, which, while useful for identifying general trends and correlations, often lack the granularity required to understand the dynamic and context‐specific nature of real‐life social interactions. Such datasets typically depend on self‐reported perceptions or large‐scale aggregated indices, making it difficult to measure the quality, immediacy, and mutuality of peer‐to‐peer exchanges.

In contrast, the present study directly mapped real‐world dyadic peer relationships using a name‐generator nomination method among first‐year university students within a single academic cohort. This approach enables the capture of immediate, reciprocal social ties embedded in daily interactions, something that traditional big data methods cannot adequately reflect. By situating social network analysis (SNA) within a naturally bounded group of late adolescents transitioning into early adulthood, this study offers ecologically valid insights into how peer connectedness manifests in emerging adulthood and how it relates to mental health outcomes.

Centrality measures reflect distinct aspects of network position that provide a more nuanced understanding of how individuals are embedded within their peer networks. In this study, we employed four commonly used centrality indicators: degree, closeness, betweenness, and eigenvector centrality, each capturing different dimensions of peer influence and network integration. Degree centrality measures the number of direct peer connections, indicating an individual's overall level of social activity or visibility (Mojzisch et al. 2021). Closeness centrality reflects how quickly a person can reach others within the network, associated with access to information or support (Freeman 2004). Betweenness centrality identifies individuals who act as bridges between otherwise unconnected peers, reflecting their potential to control information flow or mediate social tensions (Son et al. 2021). Lastly, eigenvector centrality captures influence through both direct and indirect connections by giving greater weight to ties with highly connected peers (Pan et al. 2020).

By applying these metrics, the present study goes beyond merely identifying who is connected to whom and instead explores how different forms of social embeddedness within a peer network relate to psychological well‐being in late adolescence.

Thus, this study aims to directly construct and analyze the social networks of first‐year university students, a late adolescent group forming new social connections, to investigate the impact of social network strength on mental health.

## 2 Methods

### 2.1 Participants and Study Period

This study initially recruited 120 first‐year students enrolled at S University, located in City A, Chungcheongnam‐do, South Korea. Among them, 12 students (10%) either declined to participate or submitted incomplete responses and were therefore excluded from the final analysis. As a result, 108 students (90%) who provided informed consent and complete data were included in the final analysis. Data were collected via a structured survey administered between August 1 and October 1, 2024.

Participants were selected based on the criteria established by Son et al. (2021). The inclusion criteria required participants to meet the following conditions:

‐ have no diagnosis or history of intellectual disability or organic mental disorders
‐ voluntarily consent to participate in the study

The exclusion criteria included individuals who met the following conditions:

Antipsychotic medications may impair emotional expressivity and social cognitive functioning, which are central to understanding peer network structure (Green et al. 2008), and thus, participants using such medications were excluded

‐ had visual or auditory impairments that could affect assessment
‐ had neurological or orthopedic conditions affecting body structure and function

All participants met the inclusion and exclusion criteria and provided informed consent, with the option to withdraw at any time. To protect participants' privacy within the context of small‐network analysis, all identifiable network data were anonymized immediately after encoding, and individual responses were fully de‐identified prior to analysis.

The required sample size was determined using G*Power 3.1.9.4, based on effect sizes reported in previous research examining the association between close interpersonal relationships and psychological well‐being among university students. In particular, Graham and Barnfield (2021) found a strong positive correlation between intimacy with a significant other, representing close, high‐quality interpersonal connections and psychological well‐being (*r* = 0.58) in late adolescents, a developmental stage characterized by the rapid expansion and deepening of meaningful social relationships. Using this effect size, with a significance level of 0.05 and a statistical power of 0.90, a two‐tailed test indicated that a minimum of 23 participants was required for the correlation analysis. To ensure study reliability and account for potential attrition, a total of 120 university students were initially recruited.

The study was conducted from July 1 to December 1, 2024, and received approval from the Soonchunhyang University Institutional Review Board (IRB No. 1040875‐202404‐SB‐036).

### 2.2 Research Procedure

To obtain consent for participation, the principal investigator visited the institution in person and provided an explanation of the study's purpose and objectives in a comfortable and stable environment within a spacious lecture hall. To facilitate understanding, visual aids were used during the explanation.

Participants who fully understood the purpose, objectives, and methods of the study and voluntarily agreed to participate provided written informed consent. Those who completed the survey received a small compensation.

Participants individually responded to the survey, and the evaluation process was conducted by one principal investigator and one assistant researcher. The principal investigator explained the survey and guided the process, while the assistant researcher recorded and scored the responses.

### 2.3 Social Network Analysis

In this study, social network strength was set as the unit of analysis, and data were collected through structured surveys. Participants evaluated their interpersonal relationships by rating the level of closeness with their peers on a 1–10 scale. The collected data were encoded using the edge list coding method in Excel to assess the closeness levels within friendship networks. A data preprocessing stage was conducted to remove unnecessary information, followed by network modeling to structure the dataset for analysis.

Since the study focused on friendship‐based networks within a single university cohort, a 1‐mode network was constructed to represent direct student‐to‐student ties. A 1‐mode network enables a clear examination of mutual relationships and structural positions among actors, which aligns with the objective of identifying central individuals and cohesive subgroups. In contrast, constructing a 2‐mode network (e.g., connecting individuals to shared affiliations or external entities such as clubs, family, or activities) would introduce additional layers that could obscure the core peer dynamics specific to university students (Wasserman and Faust 1994). Therefore, the 1‐mode approach was deemed more appropriate for capturing direct interpersonal connectedness in a bounded cohort of late adolescents.

Utilizing a 1‐mode network allowed for the analysis of centrality, cohesion, and subgroup structures within the network (Son et al. 2021). This approach also enabled the analysis of relationship patterns and social ties, facilitating a more precise measurement of the strength and density of social connections within the same network.

Through this framework, the study conducted an in‐depth analysis of the structural characteristics of friendship networks, focusing on the significance of relationship strength within social networks. The collected data were analyzed using SNA, focusing on centrality indicators as key measures (Table 1). Based on Freeman (2004), four centrality measures were utilized: degree centrality, closeness centrality, betweenness centrality, and eigenvector centrality. In the centrality analysis, relationship strength was weighted based on the frequency of interactions and the level of closeness, with closeness measured on a 1–10 scale, where higher scores indicated stronger interpersonal connections.

**TABLE 1:** Classification of network analysis index.

Classification	Analysis methods	Index	Content
Central structure analysis	Network/node level	Degree centrality	The extent to which a node in a network is directly connected to other nodes. It quantifies the number of direct connections a node has within the network.
		Closeness centrality	A concept emphasizing the distance between nodes in an entire network, measuring the centrality of a node by considering both direct and indirect connections to assess its overall accessibility within the network.
		Betweeness centrality	The role of a node in controlling or mediating relationships between all other nodes in the network, indicating its influence in facilitating or restricting interactions.
		Eigenvector centrality	A metric that reflects the centrality of a node by incorporating the centrality of its connected nodes, assigning greater importance to nodes that are linked to other highly influential nodes.

### 2.4 Research Instruments

#### 2.4.1 Structured Questionnaire

This study utilized a structured questionnaire consisting of 18 items, including six questions assessing general characteristics of university students, six questions examining social network structures, and six questions evaluating mental health status. To analyze social network characteristics, the study employed the peer nomination method, based on the works of Moreno (1953) and Son et al. (2021), in which participants were asked to list a fixed number of friends to collect social network data.

The peer nomination method was implemented through an open‐ended question: “Please write the names of up to three students in your department with whom you would most like to engage in leisure activities.” To quantify relationship strength and connection weight, participants also rated their perceived level of closeness with the nominated peers. The survey was conducted using a self‐administered open‐ended format, requiring participants to list up to three friends, ranking them in order of preference, with the first name representing the closest peer. If a participant was unable to list three friends, they were instructed to write “none” in the respective fields. However, friends listed from outside the department were excluded from the SNA.

#### 2.4.2 Ideation Scale

To assess mental health, this study adopted the Korean version of the Suicidal Ideation Scale, a self‐report measure developed by Kim and Jang (2020), which includes 18 items: six items measuring hopelessness, six items measuring depression, and six items assessing suicidal ideation. This study specifically utilized the hopelessness and depression subscales. Higher total scores indicate poorer mental health. The scale consists of self‐report Likert‐type items ranging from 1 (“*Strongly Disagree*”) to 7 (“*Strongly Agree*”), with response options including “*Strongly Disagree*” (1), “*Disagree*” (2), “*Somewhat Disagree*” (3), “*Neutral*” (4), “*Somewhat Agree*” (5), “*Agree*” (6), and “*Strongly Agree*” (7). The internal consistency was acceptable, with Cronbach's alpha coefficients of 0.82 for hopelessness and 0.87 for depression. When the items from both subscales were combined to compute a total score, the Cronbach's alpha was 0.78, indicating acceptable internal consistency for the combined construct as well.

#### 2.4.3 Data Analysis

Social network data were analyzed using NetMiner 4.0 (Cyram Co.), and the output was exported and coded for statistical analysis using SPSS WIN 27.0. Descriptive statistics were employed to summarize the general characteristics of the participants.

To examine the relationships between social network centrality measured and mental health variables, a series of Multiple Regression Quadratic Assignment Procedure (MRQAP) analyses were conducted, rather than a traditional hierarchical block‐entry regression. Each MRQAP model was run independently for a specific centrality measure, beginning with in‐ and out‐degree centrality, followed by in‐ and out‐closeness centrality, betweenness centrality, and eigenvector centrality. In each analysis, mental health indicators were entered as dependent variables.

This sequential modeling approach allowed for the isolation of effects associated with each network position. All statistical significance levels were set at *α* = 0.05. The regression results for each centrality metric were reported independently and are presented in sequential order in the results tables.

Portions of the language in Section 2 were refined using ChatGPT (OpenAI, San Francisco, CA, USA) to improve clarity and readability. No content, analysis, or interpretation of results was generated by AI, and all intellectual contributions were made by the authors.

## 3 Results

### 3.1 Demographic Characteristics Analysis

The demographic characteristics of the participants in this study are as follows (Table 2). Among the late adolescent participants, 39 were male and 69 were female, with an average age of 19.41 years for males and 19.38 years for females. In terms of academic performance, 3 participants were in the lower tier, 11 in the lower‐middle tier, 34 in the middle tier, 17 in the upper‐middle tier, and 4 in the upper tier.

**TABLE 2:** Results of general characteristics (*n* = 108).

Classification		*n*
Sex	Male	39
	Female	69
Age (average)	Male	19.41
	Female	19.38
Academic achievement	Bottom tier	3
	Lower‐middle tier	11
	Middle tier	34
	Upper‐middle tier	17
	Top tier	4
Life satisfaction	Very dissatisfied	1
	Dissatisfied	2
	Neutral	29
	Satisfied	28
	Very satisfied	10
Mental health status	Very unhealthy	2
	Unhealthy	3
	Fair	22
	Quite healthy	27
	Very healthy	15

Regarding life satisfaction, 1 participant reported being very dissatisfied, 2 were dissatisfied, 29 rated their satisfaction as neutral, 28 were satisfied, and 10 were very satisfied. In terms of mental health status, 2 participants reported being very unhealthy, 3 reported being unhealthy, 22 indicated a fair state, 27 considered themselves somewhat healthy, and 15 rated themselves as very healthy.

### 3.2 Quadratic Assignment Procedure (QAP) Correlation Analysis

The results of the QAP correlation analysis examining the relationship between social network strength and mental health are presented in Table 3.

**TABLE 3:** Results of QAP correlation analysis.

Network of intimacy	Hopelessness	Depression	Total
In‐degree centrality	−0.167(0.102)	−0.233^*^(0.02)	−0.217^*^(0.031)
Out‐degree centrality	−0.004(0.967)	−0.069(0.509)	−0.044(0.675)
In‐closeness centrality	−0.252^*^(0.011)	−0.256^**^(0.01)	−0.27^**^(0.006)
Out‐closeness centrality	−0.028(0.79)	0.062(0.55)	0.025(0.808)
Betweenness centrality	−0.113(0.277)	−0.076(0.468)	−0.097(0.351)
Eigenvector centrality	−0.194(0.056)	−0.291^**^(0.003)	−0.265^**^(0.008)

* *p*<0.05.

^**^ *p*<0.01.

For hopelessness, a statistically significant negative correlation was found with in‐closeness centrality (*r* = −0.252, *p* < 0.05), indicating that higher in‐closeness centrality values were associated with lower levels of hopelessness.

For depression, significant negative correlations were observed with in‐degree centrality (*r* = −0.233, *p* < 0.05), in‐closeness centrality (*r* = −0.256, *p* < 0.05), and eigenvector centrality (*r* = −0.291, *p* < 0.01), suggesting that higher values of these centrality measures were linked to lower levels of depression.

Finally, for overall mental health issues, which include both hopelessness and depression, significant negative correlations were found with in‐degree centrality (*r* = −0.217, *p* < 0.05), in‐closeness centrality (*r* = −0.270, *p* < 0.01), and eigenvector centrality (*r* = −0.265, *p* < 0.01), indicating that higher levels of these centrality measures were associated with fewer mental health problems.

### 3.3 QAP Regression Analysis

The results of the QAP regression analysis, which examined the impact of social network strength on mental health, are presented in Table 4.

**TABLE 4:** Results of QAP regression analysis.

Network of intimacy	Hopelessness	Depression	Total
In‐degree centrality	0.84	0.323	1.163
Out‐degree centrality	0.999	−2.481	−1.482
In‐closeness centrality	−21.706	−13.174	−34.88
Out‐closeness centrality	3.718	49.533^*^	53.251
Betweenness centrality	13.508	−3.402	10.106
Eigenvector centrality	−3.459	−14.288	−17.747
Constant	13.573	17.383	30.956
*F*	1.157(0.338)	2.423^*^(0.033)	1.836(0.1)
*R* ^2^	0.074	0.143	0.112

* *p *< 0.05.

For the dependent variable hopelessness, the model had an adjusted *R* ^2^ of 0.074, but it was not statistically significant (*F* = 1.157, *p* = 0.338).

For the dependent variable depression, the model had an adjusted *R* ^2^ of 0.143 and was statistically significant (*F* = 2.423, *p* < 0.05). Additionally, all collinearity statistics indicated that there were no multicollinearity issues, as Variance Inflation Factor (VIF) values were below 10 and tolerance values exceeded 0.1.

For the overall mental health measure, which includes both hopelessness and depression, the model had an adjusted *R* ^2^ of 0.112 but was not statistically significant (*F* = 1.836, *p* = 0.1).

The analysis revealed that out‐closeness centrality significantly influenced depression, indicating that a 1% increase in out‐closeness centrality corresponded to an increase of 49.533 in depression levels. This suggests that greater out‐closeness centrality is associated with higher levels of depression.

## 4 Discussion

This study aimed to examine the relationship between social interactions within friendship networks and mental health in late adolescence and to investigate the impact of social network strength on mental well‐being. The findings suggest that individuals with higher in‐closeness centrality tend to experience lower levels of hopelessness, while those with greater in‐degree centrality, in‐closeness centrality, and eigenvector centrality exhibit lower levels of depression, highlighting the potential protective effects of being well‐connected within a social network. However, the regression analysis indicates that while these associations exist, hopelessness does not reach statistical significance, suggesting that its relationship with network centrality may be influenced by other factors. Notably, the significant positive association between out‐closeness centrality and depression implies that individuals who need to reach out to more distant network members may experience higher levels of depressive symptoms, potentially due to weaker immediate social support or increased effort required to maintain connections. This finding underscores the complexity of social network structures in shaping mental health outcomes.

These findings confirm the significant relationship between social network interactions and mental health in late adolescence. Specifically, the QAP correlation analysis revealed that among all centrality measures, only in‐closeness centrality showed a significant negative association with hopelessness. Unlike degree centrality, which merely quantifies the number of ties without addressing their quality or reciprocity (Valente et al. 2008), or out‐closeness centrality, which reflects the speed at which an individual can reach others but offers limited emotional buffering (Ge and Zhang 2024), in‐Closeness centrality emphasizes how quickly and easily others can reach a given individual. This form of accessibility fosters reciprocal and frequent contact, thereby ensuring that emotional support can be mobilized rapidly. Such immediacy is particularly salient to hopelessness, as it mitigates social isolation and helplessness core elements of the construct and critical precursors of suicidal ideation (Ge and Zhang 2024).

Prior studies have emphasized that network positions enabling rapid inbound connections foster a sense of embeddedness and belonging, which are critical for emotional resilience in vulnerable populations (Pan et al. 2020). These findings highlight that hopelessness is more sensitive to the perception that others are both willing and able to reach out in times of need, making in‐closeness a critical protective factor. While in‐closeness was most strongly associated with reduced hopelessness, its link to depression may involve mechanisms that partially overlap with, yet also differ from, those influencing hopelessness. In the Korean university context, the immediacy of inbound access can help counteract the acute isolation and helplessness that drive hopelessness. By contrast, depression often shaped by cumulative and persistent stressors may require not only quick access but also sustained, high‐quality interactions to ensure long‐term emotional stability.

Network‐based interventions should prioritize enhancing the efficiency of social pathways for at‐risk students by fostering reciprocal, high‐quality connections rather than merely expanding network size. Representative strategies include structured peer mentoring programs, small‐group collaborative learning teams, and bridge‐building activities facilitated by trained peer leaders. Implemented in a systematic format, such as biweekly peer meetings with participation tracking and brief outcome assessments, these initiatives can effectively shorten the social distance between isolated individuals and central nodes, thereby improving access to emotional and informational resources while reducing psychological disconnection.

While in‐closeness centrality uniquely buffered against hopelessness, the analysis also revealed that in‐degree centrality, in‐closeness centrality, and eigenvector centrality were each significantly and negatively associated with depression, reflecting distinct structural advantages within the network. In‐degree centrality, which measures the number of incoming connections, suggests that students approached by more peers often receive greater perceived social support and validation, functioning as key channels for both emotional and instrumental resources (Valente et al. 2008).

In the Korean university context, incoming ties carry heightened psychological significance. Being sought out by peers serves as a visible marker of trust, recognition, and inclusion within tightly knit academic or extracurricular communities (Son et al. 2021). Such relationships are frequently developed through shared academic challenges, participation in student clubs, or informal study groups, creating stable foundations of mutual obligation and sustained emotional exchange that protect against depressive symptoms (Choi 2012). These findings are consistent with prior research demonstrating that higher in‐degree centrality in university students’ interpersonal networks amplifies protective effects on mental health, as rapid and reciprocal connections enhance a sense of belonging and psychological security (Son et al. 2021).

To implement these findings, campus‐based interventions should intentionally increase the visibility and accessibility of at‐risk students within their peer networks. Structured approaches, such as welcome‐group matching programs, peer leader–led outreach events, and targeted mentorship pairings, can ensure that vulnerable students are integrated into supportive social clusters. When designed with clear participation guidelines and monitored outcomes (e.g., participation rates, changes in social network measures, reductions in depressive or hopelessness scores), these interventions can promote timely peer support, reduce emotional isolation, and strengthen both individual resilience and overall network cohesion.

In addition to the cultural significance of in‐degree centrality, in‐closeness centrality was also significantly and negatively correlated with depression in the correlation analysis. In the Korean university context, where proactive peer engagement and reciprocal care are highly valued, the immediacy of inbound access plays a critical role in emotional well‐being. Many students may hesitate to initiate help‐seeking behaviors due to modesty, fear of burdening others, or social norms prioritizing harmony (Lee et al. 2008). In such cases, the ability for peers to reach out quickly enables timely emotional and informational support, alleviating acute feelings of isolation and buffering against depressive symptoms (Bae and Lee 2023). This dynamic resonates with the Korean concept of Jeong, where consistent, caring interactions strengthen trust, solidarity, and a shared sense of responsibility within peer groups.

Eigenvector centrality also holds a unique meaning in the hierarchical and relationship‐oriented culture of Korean universities. Connections with student leaders, respected senior mentors, or socially central figures not only open pathways to valuable academic resources, internship opportunities, and professional networks but also serve as symbolic affirmations of social standing and group membership (Kwon 2017; Kang and Lim 2008). Such recognition can reinforce self‐worth, resilience, and a sense of belonging, thereby helping to mitigate depressive symptoms. However, consistent with Pan et al. (2020), such ties do not inherently guarantee emotional closeness and, in some cases, may introduce social comparison pressures or role‐related stress, particularly in competitive academic environments.

Recognizing these cultural dynamics, network‐based interventions should target multiple centrality dimensions. For in‐closeness, network‐based interventions could focus on creating structured and intentional opportunities that enhance the efficiency and immediacy of social pathways for at‐risk students. Examples include peer‐led outreach circles that operate on a scheduled basis, structured check‐in systems (e.g., biweekly meetings with participation tracking), and small‐group collaborative learning teams or project‐based cohorts that encourage sustained, reciprocal interaction. Additionally, bridge‐building activities facilitated by trained peer leaders can help connect socially peripheral students to more central and resourceful peers.

For eigenvector centrality, programs could strategically connect at‐risk students with socially influential peers through ambassador‐style mentorship, leadership‐led events, and collaborative participation in high‐visibility campus activities. When implemented with clear participation guidelines and monitored for both quantitative (e.g., network measure changes, reduction in depression scores) and qualitative (e.g., perceived support, relationship quality) outcomes, these interventions can leverage culturally embedded relationship patterns to enhance psychological security, inclusion, and resilience among vulnerable students.

The regression analysis revealed that out‐closeness centrality characterized by broad but potentially shallow connections was linked to increased depressive symptoms. Furthermore, the results of the QAP regression analysis identified out‐closeness centrality as a significant predictor of depression, indicating that individuals with higher out‐closeness centrality tend to experience greater levels of depressive symptoms. Out‐closeness centrality reflects the extent to which an individual can rapidly access others within a network (Scott 2000; Wasserman and Faust 1994). In the context of friendship networks, adolescents with high out‐closeness centrality occupy structurally advantageous positions that facilitate efficient and broad interactions. However, this structural reach may come at a psychological cost. Specifically, when adolescents are sensitive to peer evaluation, the increased social visibility and relational demands associated with high centrality can impose emotional and temporal burdens, potentially leading to psychological exhaustion and heightened depressive symptoms (Falci and McNeely 2009; Ueno 2005).

These findings are consistent with prior research emphasizing that elevated out‐closeness centrality may exacerbate stress rather than provide support, especially when interpersonal ties lack emotional depth or reciprocity (Bringmann et al. 2019; Valente et al. 2008). Bringmann and colleagues, in particular, argue that the energetic cost of maintaining a wide array of weak ties can exceed emotional resilience, ultimately impairing mental health. This aligns with developmental studies showing that adolescents in highly central positions within friendship networks, especially those with heightened fear of negative evaluation, face increased psychological vulnerability due to the pressures of maintaining broad social ties (Kornienko and Santos 2014). Overall, these findings underscore the need to consider both the structural and qualitative dimensions of peer relationships when interpreting centrality effects. While out‐closeness may signify broad reach, it does not guarantee emotional connectedness.

Taken together, these findings highlight that while out‐closeness centrality reflects a structurally advantageous position, it may also be associated with heightened depressive symptoms under certain conditions. This relationship can be more fully understood and, in the Korean context, potentially intensified by considering the unique developmental and sociocultural characteristics of late adolescence in Korea. From a developmental perspective, this negative association can be understood within the socio‐environmental contexts of late adolescence. In Korea, most adolescents before entering university live in highly structured, academically driven environments focused on college entrance preparation (Park et al. 2012). Upon admission, they gain autonomy and encounter diverse sociocultural environments (Kim and Seo 2015), a transition that can challenge adaptation and lead to anxiety, depression, or psychosomatic symptoms, especially for first‐year students still developing psychosocial skills (Yoo 2019).

In this transitional stage, students with high out‐closeness centrality have more opportunities for positive exchanges but are also more exposed to negative influences and social pressures. Elevated centrality increases relational demands and self‐regulation strain, which can result in emotional exhaustion and depression effects that are amplified when coping resources are still maturing. In Korea's competitive, community‐oriented culture, peer approval is central to psychological security, fostering conformity to maintain group harmony (Roh et al. 2018; Choi et al. 2015). Students in socially central positions may feel heightened pressure to avoid rejection, leading to emotional suppression and compliance with group expectations, consistent with Baumeister et al. (2005) assertion that social exclusion impairs self‐regulation and increases depression risk.

The rapid expansion of online communication further intensifies these dynamics. In Korea, high internet penetration and smartphone use facilitate constant peer connection via SNS, YouTube, and instant messaging. High out‐closeness centrality in digital contexts entails frequent real‐time exchanges of information and value judgments, fostering continual self‐monitoring and comparison. Such dynamics can increase emotional fatigue and depressive symptoms, with negative online interactions linked to self‐harm and suicidal ideation (Abi‐Jaoude et al. 2020; John et al. 2018). Fear of Missing Out (FoMO) can make relationship maintenance a persistent stressor (Vogel et al. 2006).

These network effects are further shaped by relationship‐centered collectivism in Korea, which emphasizes familism, kinship solidarity, benevolent relationships, and community orientation, with group‐centeredness and benevolent human relations being particularly salient (You and Shim 2013). While group‐centeredness offers psychological security, it also imposes conformity pressures. Unlike Western collectivism, which prioritizes group goals over individual goals, Korean collectivism pragmatically combines collective benefit with personal gain. Benevolent human relations characterized by deep emotional bonds, mutual concern, and harmony are considered prerequisites even for transactional relationships (You and Shim 2013) and often originate from shared backgrounds such as hometowns or schools, later deepening through emotional investment (Choi 2000). Within such tightly connected networks, high out‐closeness centrality entails managing numerous emotionally charged relationships while meeting expectations of benevolence, reciprocity, and harmony. This relational and emotional labor heightens cognitive and affective demands, increasing the risk of emotional exhaustion and depression. Relationship‐centered collectivism may therefore moderate the link between out‐closeness centrality and depression, with stronger collectivistic values amplifying the negative association.

Network‐based interventions could include structured peer mentoring programs, welcome‐group matching initiatives, or campus‐based activities that foster reciprocal engagement for enhancing in‐degree centrality; small‐group collaborative learning teams and peer exchange circles for reinforcing in‐closeness; and leadership‐led outreach programs or ambassador‐style peer support networks to strengthen beneficial aspects of eigenvector centrality. Together, these approaches can increase the visibility, accessibility, and integration of at‐risk students, promoting timely support and fostering positive mental health outcomes.

This study systematically analyzed the impact of social network centrality on mental health in late adolescence, elucidating the dual nature of centrality, which has been largely overlooked in previous research. While prior studies have predominantly emphasized the positive role of centrality in enhancing social support and psychological stability, this study provides empirical evidence that out‐closeness centrality may, in fact, contribute to increased depression. This finding suggests that the burden of maintaining distant social connections and the lack of immediate emotional support may exacerbate psychological distress, indicating that social integration does not necessarily yield positive mental health outcomes. Furthermore, although the beneficial role of in‐closeness centrality has been acknowledged in previous research, this study is among the few to empirically establish its direct association with reduced hopelessness, underscoring the necessity of strengthening social relationships as a suicide prevention strategy. Given these potential risks, targeted interventions are warranted to mitigate the adverse effects of high out‐closeness centrality on mental health. For these individuals, social skills workshops and psychoeducation sessions focused on setting boundaries, managing social expectations, and identifying emotionally meaningful relationships could be helpful. Moreover, small‐group cohesion training may redirect students toward deeper interpersonal engagement rather than superficial interactions.

Additionally, this study challenges the conventional assumption that eigenvector centrality is inherently advantageous, demonstrating that while it reflects connections with influential network members, it does not inherently ensure relationship quality and may, in some cases, impose psychological burdens. This finding offers a novel perspective distinct from prior studies. In conclusion, this study delineates the protective and adverse effects of social network centrality on mental health, emphasizing the importance of interventions that go beyond mere network expansion to focus on qualitative relationship improvement and alleviation of psychological burdens. These findings provide critical implications for the development of mental health policies and intervention programs.

First, the study employed a cross‐sectional design, which inherently limits the ability to draw causal inferences between network centrality and mental health outcomes. Because both variables were measured at a single point in time, it is unclear whether certain network positions lead to changes in mental health or whether individuals with particular mental health profiles gravitate toward specific social roles. Future research should adopt longitudinal designs to disentangle directionality, clarify causal mechanisms, and track the evolving impact of network centrality over time. Second, this study was conducted with a relatively small sample of Korean students, limiting its external validity and generalizability to broader adolescent populations and across different cultural contexts. Additionally, it did not account for the qualitative aspects of relationships, which are crucial in understanding the link between social networks and mental health. However, given that late adolescents worldwide share similar developmental characteristics, the findings may still provide meaningful insights into how social network structures influence mental health across different settings. Future research should address these limitations by including larger and more diverse samples across multiple cultural backgrounds, adopting multilayered analyses that integrate both quantitative and qualitative relationship characteristics, and implementing longitudinal study designs to better capture the dynamic interactions between social networks and mental health over time.

## Author Contributions

**Jin Seok Oh**: conceptualization, investigation, writing–original draft, visualization, methodology, writing–review and editing, data curation. **Jong Gab Ho**: investigation, writing–review and editing, data curation. **Jin‐Hyuck Park**: conceptualization, writing–review and editing, project administration

## Ethics Statement

The study was conducted from July 1 to December 1, 2024, and received approval from the Soonchunhyang University Institutional Review Board (IRB No. 1040875‐202404‐SB‐036).

## Consent

Written informed consent was obtained from all participants prior to study enrollment.

## Conflicts of Interest

The authors declare no conflicts of interest.

## Peer Review

The peer review history for this article is available at https://publons.com/publon/10.1002/brb3.70910

## Data Availability

The datasets generated and/or analyzed during the current study are from University students. Thus, after obtaining the subjects’ permission for this study, they are available from the corresponding author upon reasonable request.
